# Characteristics of Patients Co-infected with Severe Acute Respiratory Syndrome Coronavirus 2 and Dengue Virus, Buenos Aires, Argentina, March–June 2020

**DOI:** 10.3201/eid2702.203439

**Published:** 2021-02

**Authors:** Lucila M. Carosella, Daniel Pryluka, Aldo Maranzana, Laura Barcan, Rosana Cuini, Cristina Freuler, Alfredo Martinez, Tomás Rivero Equiza, Carolina Rodriguez Peria, Diego Yahni, Martin E. Stryjewski, for the COVIDENGUE Study Group

**Affiliations:** Centro de Educación Médica e Investigaciones Clínicas, Buenos Aires, Argentina (L.M. Carosella, A. Martinez, T. Rivero Equiza, M.E. Stryjewski);; Sanatorio Otamendi, Buenos Aires (D. Pryluka);; General Hospital de Agudos Parmenio Piñero Gobierno de la Ciudad, Buenos Aires (A. Maranzana);; Hospital Italiano de Buenos Aires, Buenos Aires (L. Barcan);; Hospital de Agudos Dr. Teodoro Álvarez, Buenos Aires (R. Cuini);; Hospital Alemán, Buenos Aires (C. Freuler);; Sanatorio de Los Arcos, Buenos Aires (C. Rodriguez Peria),; Sanatorio Mutual del Transporte Automotor, Buenos Aires (D. Yahni)

**Keywords:** Severe acute respiratory syndrome coronavirus 2, SARS-CoV-2, dengue virus, dengue fever, coronaviruses, viruses, coronavirus disease, COVID-19, respiratory infections, co-infection, zoonoses, Buenos Aires, Argentina

## Abstract

An epidemic of dengue virus and severe acute respiratory syndrome coronavirus 2 (SARS-CoV-2) co-infections occurred in Argentina during 2020. We describe the clinical characteristics and outcomes in a cohort of patients hospitalized because of co-infection. We retrospectively identified 13 patients from different hospitals in Buenos Aires who had confirmed infection with SARS-CoV-2 and dengue virus and obtained clinical and laboratory data from clinical records. All patients had febrile disease when hospitalized. Headache was a common symptom. A total of 8 patients had respiratory symptoms, 5 had pneumonia, and 3 had rash. Nearly all patients had lymphopenia when hospitalized. No patients were admitted to an intensive care unit or died during follow up. Co-infection with SARS-CoV-2 and dengue virus can occur in patients living in areas in which both viruses are epidemic. The outcome of these patients did not seem to be worse than those having either SARS-CoV-2 or dengue infection alone.

Severe acute respiratory syndrome coronavirus 2 (SARS-CoV-2), which produces coronavirus disease (COVID-19), and dengue caused an epidemic in Argentina during 2020. During March 3–October 25, 2020, a total of 1,090,589 confirmed cases of infection with SARS-CoV-2 were reported in this country. Of these cases, 143,990 were reported in Ciudad Autónoma de Buenos Aires (*1*). During January 4–June 13, 2020, there were ≈7,300 confirmed cases of dengue virus infection in this city (*2*).

Although co-infection with these 2 virus is a major concern, it has only been reported in individual patients (*3*–*5*). Information from cohorts of co-infected patients is still lacking. We describe the clinical characteristics and outcomes in a cohort of patients co-infected with SARS-CoV-2 and dengue virus in Buenos Aires.

## Methods

Using a network of colleagues (COVIDENGUE Study Group), we retrospectively identified patients co-infected with SARS-CoV-2 and dengue virus during March–June 2020. Seven sites were in Buenos Aires, and an additional site was in the surrounding area. Through June 30, 2020, healthcare admission was mandated in Argentina for any patient with confirmed COVID-19. Therefore, all patients had complete information regarding signs and symptoms at hospitalization, as well as their hospital course. Clinical data were obtained in a predesigned clinical report form by reviewing medical records.

Infection with SARS-CoV-2 was diagnosed by using real-time PCR of nasopharyngeal swab specimens, as approved by the Ministry of Health in reference laboratories. Dengue was diagnosed by either detection of nonstructural protein 1 (NS1), real-time PCR, or serologic conversion. Severe dengue was defined by presence of respiratory distress, severe bleeding, or organ impairment. Mild COVID-19 was defined by absence of pneumonia or pneumonia without impairment in oxygenation for otherwise stable patients. Patients requiring supplemental oxygen were considered to have moderate-to-severe COVID-19. Patients were followed up for 4 weeks from their initial hospitalizations. Descriptive statistics were used. This study was approved by the Ethic Committee of Centro de Educación Médica e Investigaciones Clínicas (Buenos Aires, Argentina).

## Results

A total of 13 patients who had co-infections with SARS-CoV-2 and dengue virus were identified. Most patients were relatively young (median age 37 years), 46% were female, and 54% reported >1 concurrent condition. All patients had febrile disease at hospitalization (Table). The median duration of fever was 7 days; 9 (69%) patients had fever for >5 days. Headache was a common symptom among co-infected patients. A total of 8 patients (62%) had respiratory symptoms. Symptoms of lower respiratory tract infection were present in 4 (31%) patients; 5 (38%) patients had ground glass opacities consistent with viral pneumonia on computed tomography (CT) scans. Two patients had bilateral infiltrates on a chest radiograph or computed tomography. Rash appeared in 3 patients early in the course of the disease and before resolution of fever. Two of these patients had concomitant pneumonia and rash. Lymphopenia was observed in all but 1 patient (92%), and thrombocytopenia was observed in 46%.

**Table:** Clinical characteristics and laboratory parameters at hospitalization for 13 patients co-infected with SARS-CoV-2 and dengue virus, Buenos Aires, Argentina, March–June 2020*

Characteristics	Value
Age, y	37 (29–50)
Sex	
F	6 (46)
M	7 (54)
Concurrent condition†	7 (54)
Signs or symptoms	
Fever at hospitalization	13 (100)
Median temperature at hospitalization, °C	38.0 (38.0–38.8)
Median duration of fever, d	7 (4–9)
Cough	3 (23)
Headache	8 (61)
Myalgia	7 (54)
Rash	3 (23)
Chills	3 (23)
Dyspnea	2 (15)
Diarrhea	2 (15)
Odinophagia	2 (15)
Nasal congestion	2 (15)
Anosmia	1 (8)
Dysgeusia	1 (8)
Arthralgia	1 (8)
Nausea or vomiting	1 (8)
Pneumonia	5 (38)
Bilateral	2 (15)
Laboratory findings	
Median hematocrit, %	44 (36–46)
Median hemoglobin, g/dL	14.1 (13.0–15.0)
Median leukocytes × 10^3^ cells/μL	4.3 (2.26–7.9)
Leukopenia, <4 × 10^3^ cells/μL	4 (31)
Median lymphocyte count × 10^3^ cells/μL	0.81 (4.1–1.16)
Lymphopenia, <1.5 × 10^3^ cells/μL	12 (92)
Platelets ×10^3^/μL	172 (116–196)
<150 ×10^3^/μL	6 (46)
<100 ×10^3^/μL	3 (23)
Abnormal AST level	6 (46)
Abnormal ALT level	6 (46)
Diagnosis of dengue	
NS1 detection	8 (61)
Real-time PCR	4 (31)
Serologic conversion	1 (8)
Clinical outcomes	
Need for supplemental oxygen	1 (8)
ICU	0
Median length of stay in hospital, d	12 (10–14)
Severe dengue	0
Hospital discharge	13 (100)
Full recovery at follow-up at 4 wk	13 (100)
Rehospitalization at 4 wk	0
Death	0
Therapy for infection with SARS-CoV-2	
Lopinavir/ritonavir	3 (23)
Hydroxychloroquine	1 (8)
Antimicrobial drug therapy	6 (46)

*Values are no. (%) or median IQR. ALT, alanine aminotransferase; AST, aspartate aminotransferase, ICU, intensive care unit; IQR, interquartile range; SARS-CoV-2, severe acute respiratory syndrome coronavirus 2. †Includes obesity 3; chronic obstructive pulmonary disease 2; hypertension 2; smoking 2; diabetes 1; and cirrhosis 1.

Among patients with fever <5 days, suspicion of dengue was based on a history of recent mosquito bite (2 patients), frontal headache (3 patients), intense myalgia (3 patients), or thrombocytopenia (2 patients). All patients had a diagnosis of COVID-19 by real-time PCR of nasopharyngeal swabs specimens. Dengue was diagnosed by detection of NS1 in >50% of co-infected patients; 4 cases were diagnosed by real-time PCR of serum and 1 by serologic conversion. The patient who had serologic conversion had a first serum sample negative for IgM and IgG. One week later, a second serum sample was positive for both antibodies. All but 1 patient (92%) had mild COVID-19. No patients had severe dengue infection, required admission to an intensive care unit, or died during follow up. All patients fully recovered from their symptoms after 4 weeks.

## Discussion

This report of patients co-infected with SARS-CoV-2 and dengue virus provides several useful observations. First, in geographic areas in which both viruses are circulating, co-infections can occur. This concerning possibility, which might impose an additional burden on healthcare systems, has been reported previously (*6*). In Latin America, >3 million cases of dengue were reported during 2019 (*7*). Dengue virus circulates epidemically in Argentina, particularly in the northeastern region of this country (*8*). The most recent epidemic occurred in 2016 (*2*). In Buenos Aires, the number of cases reported during 2017–2019 was relatively low. For example, during 2018 only 151 cases were reported and during 2019 only 51 cases in this city. However, during 2020, the magnitude of the dengue epidemic in Buenos Aires surpassed case counts for the preceding 10 years. Therefore, given the current circulation of SARS-CoV-2 at high levels, a new epidemic of dengue virus during early 2021 (warm months) could substantially increase the risk for co-infections.

Second, in this scenario of concomitant circulation of SARS-CoV-2 and dengue virus, the distinction between clinical diseases among febrile patients is crucial. Certain clinical characteristics among these co-infected patients are relevant. All patients who had co-infections had fever at hospitalization, and most had fever duration for ≥5 days. Also, >50% of patients with COVID-19 did not have fever at admission (*9*). Prolonged fever has been associated with more severe disease in patients with COVID-19 (*10*). Therefore, for most patients with mild COVID-19 in this study, prolonged fever was a clinical clue for suspecting co-infection. Other than fever, headache was the most common symptom in patients with co-infection. Although headache is common in patients who have dengue infection (>90%) (*11*) it is less commonly observed in patients with COVID-19 (≈13%) (*9*). For some co-infected patients, a clinical overlap based on hallmarks of both diseases was also noted. For example, 2 patients had pneumonia, suggesting COVID-19, as well as a rash, which can be a hallmark of dengue virus infection. The duration of fever longer than expected for mild COVID-19, headache, rash, or absence of respiratory symptoms should raise the suspicion of a concomitant infection with dengue virus. Therefore, clinical suspicion based on epidemiologic grounds might alert clinicians to order tests for both viruses.

Third, all patients had favorable outcomes for both COVID-19 and dengue virus infections. There is conflictive data on the clinical outcome of co-infection with dengue and other viruses (*12*–*14*). All but 1 of our patients had mild COVID-19, and none had severe dengue. Our preliminary findings, based on limited data, do not suggest that co-infection with dengue and SARS-CoV-2 viruses worsens clinical outcomes.

Our study had several limitations. False-positive IgM results for dengue have been described for 2 patients who had COVID-19 (*15*). However, only 1 of our patients had a serologic diagnosis of dengue, and this diagnosis was based on serologic conversion for IgM and IgG. Almost all our patients had positive results for virus NS1 tests or real-time PCR of serum for dengue. Because these tests for dengue have high specificity for acute infection (*16*), a false-positive diagnosis is unlikely (*17*). Immune response was not evaluated in our study. Analyzing the immune activation for these co-infected patients would help to clarify the clinical outcomes of these patients who have simultaneous viral infections.

Finally, our data are limited by a small sample size. Our observation on the unaltered clinical course of COVID-19 concomitant with dengue infection needs to be confirmed in larger cohorts of patients, including a comparative analysis of persons infected only with SARS-CoV-2 or dengue virus, and patients who are co-infected with both viruses.

In conclusion, co-infection with SARS-CoV-2 and dengue virus can occur in patients living in areas in which both viruses are epidemic. Some clinical clues can orient physicians to suspect both diseases. Based on limited data, our study suggests that the clinical outcome of these co-infected patients may not be worse than for patients who have either SARS-CoV-2 or dengue infection alone.
